# Assessment of increased risk of arrhythmia in advanced age pregnancies

**DOI:** 10.12669/pjms.343.14313

**Published:** 2018

**Authors:** Mehmet Musa Aslan, Adem Atici

**Affiliations:** 1 Mehmet Musa Aslan, MD. Instructor, Department of Obstetrics and Gynecology, Mus State Hospital, Mus, Turkey; 2 Adem Atici, MD. Instructor, Department of Cardiology, Mus State Hospital, Mus, Turkey

**Keywords:** Advanced age, Arrhythmia, Electrocardiogram, Pregnancy, Ventricular repolarization

## Abstract

**Background and Objective:**

Cardiovascular deaths usually occur in older pregnancies and arrhythmia is the third most common cause. Our study aimed to determine whether the risk of arrhythmia increases in pregnancy with advanced age.

**Methods:**

In total, 280 pregnant women, of whom 98 were of advanced age and 182 were under 35 years of age were included in the study. The risk of arrhythmia was evaluated by calculating the electrocardiographic P-wave duration, QT interval, T peak-to-end interval, and the Tp-e/QT ratio.

**Results:**

Although there were no differences in the Tp-e interval and Tp-e/QTc ratio between the groups, the maximum QTc, minimum QTc, and QTc dispersion values were significantly higher in advanced-age pregnancies compared to the control group. In addition, the P dispersion was greater in advanced-age pregnancies. In correlation analysis, the increased dispersion of QTc and P were positively correlated with maternal age. Multiple linear regression analysis showed that QTc dispersion was independently associated with maternal age.

**Conclusion:**

Repolarization parameters increase in advanced-age pregnancies even though they remain in the normal range, which should lead to an investigation of whether this is a pathological condition.

## INTRODUCTION

Particularly in the last 20 years, the reproductive behavior of women has changed due to socio-economic improvements, longer education, and difficulties associated with finding jobs. Therefore, women started becoming pregnant at a late age.^1^ In developed countries, an increasing proportion of births are attributable to women of advanced maternal age. In Canada, the proportion of live births to women 30–39 years old has risen from 23.6% in 1982 to 45.9% in 2005.^2^ However, the advanced age of the mother has shown a relationship with decreased fertility and increased risk of still birth, preterm live birth, low-birth weight, and adverse pregnancy outcomes.^3^-^5^ Several studies have found that pregnant women over 35 years old had unfavorable pregnancy outcomes and are generally considered as an age cut-off.^1^-^5^

In a study investigating during pregnancy up until one year postpartum, 22.2% of maternal deaths were found to be from cardiovascular disease and women with cardiovascular mortality were likely older and die during postpartum. The most frequent etiology was cardiomyopathy, while the arrhythmic deaths were the third most common cause.^6^

Although advanced maternal age is well-known to account for an increase in mortality and morbidity during pregnancy, in obstetric problems and for a higher risk of miscarriage, stillbirth, and infant death, no study has evaluated the risk of arrhythmia, which is a major cause of cardiovascular disease in maternal death. Our study aimed to determine the risk of arrhythmia by calculating the electrocardiographic P-wave duration, QT interval, T peak-to-end interval, and the Tp-e/QT ratio in pregnant women over35 years old.

## METHODS

This cross-sectional study was approved by the local ethics committee of Mus Alparslan University (no: 79236777-050.04). All consecutive advanced age (≥35 years) and <35 years (as a control group) in the last trimester according to last menstrual period and ultrasonographic measurements with asymptomatic nulliparous pregnant women who were admitted to the Department of Obstetrics and Gynecology of Mus State Hospital were considered eligible for the study. In total, 280 pregnant women, of whom 98 were of advanced age and 182 were under 35 years old, were included.

Those with multiple pregnancies, hypertension, diabetes mellitus, gestational diabetes, pre-eclampsia, eclampsia, previous history of pregnancy-induced hypertension, family history, presence of coronary heart or significant valvular heart disease, decompensated heart failure, any immunologic-rheumatologic disease, abnormal renal, hepatic, or thyroid function tests, atrial fibrillation, complete or incomplete bundle branch block, ST–T abnormalities, the use of any drugs that could affect Tp-e or QT interval, U waves or negative T waves on ECG, and electrolyte imbalances were excluded from the study.

A 12-lead ECG (AT-102, Schiller AG, Baar, Switzerland) was recorded for each woman only once at a point in 3^rd^ trimester at rest while in the supine position. Recordings were acquired at a paper speed of 50 mm/s, with 1 mV/cm standardization. We improved our accuracy using calipers and magnifying lenses. The onset of the P wave was defined as the first atrial deflection from the isoelectric line, and the offset was the return of the atrial signal to the baseline. The maximum and minimum P wave duration were measured and their differences were defined as the P dispersion. The QT interval was measured from the beginning of the QRS complex to the end of the T wave and corrected for the heart rate using the Bazett formula:

cQT=QT√(R–R interval)

The Tp-e interval was defined as the interval between the peak and end of the T wave, measurements of the Tp-e interval were performed from precordial leads, and the Tp-e/QTc ratio was calculated from these measurements.

### Statistical Analysis

Statistical analysis was performed using SPSS 21 (SPSS Inc., Chicago, Illinois). In the interim statistical analysis of the study, the sample size was calculated according to the QTc interval and a sample size of 132 (66 per group) patients would be required with 80% power and the conventional 2-sided type 1 error of 5%. Data were tested for normality of distribution using the Kolmogorov-Smirnov test. Continuous variables were presented as means followed by the standard deviation and categorical variables as frequencies and percentages. Continuous variables between the two groups were compared using Student’s t test for normally distributed data and the Mann–Whitney U test for data that was not normally distributed. Categorical parameters were evaluated by chi-squared (χ^2^) test. Pearson rank tests were used to indicate the correlation of maternal age with QTc duration, Tp-e interval, and Tp-e/QTc ratio. Multivariate linear regression analysis was performed to determine the predictors of QTc dispersion. A two-tailed P ≤ 0.05 was considered significant.

## RESULTS

All pregnant women were nulliparous and in their third trimester (28–40 weeks). The obstetric and demographic characteristics of the groups were as presented in Table-I. The women in Group-1 were older as expected. Women were at a further gestational week and the body mass index (BMI) was naturally increased in Group-1. Although blood pressure was higher in pregnant women of advanced age, their blood pressure was in the normal range. The laboratory characteristics of advanced age pregnant women and the control group upon initial admission are as presented in Table-II.

**Table-I:** Characteristics of the study population.

	Group-1 (≥35 years)	Group-2 (<35 years)	P-value
Maternal age, years	38.5± 2.57	23.5± 4.16	<0.001
Gestational week	34.7±3.5	33.4± 3.4	0.008
BMI, kg/m²	30.4±4.9	28.2±4.3	<0.001
Heart rate, bpm	90.7±14.8	89.9±15.5	0.68
Systolic BP, mmHg	117.4±9.6	112.4±10.2	<0.001
Diastolic BP, mmHg	70.8±8.6	64.6±8.6	<0.001

BMI: Body Mass Index, bpm: beats per minute, BP: Blood pressure

**Table-II:** Laboratory tests results of the study population at assessment.

	Group-1 (≥35 years)	Group-2 (<35 years)	P-value
Hemoglobin (g/dl)	11.8±1.3	11.8±1.2	0.84
Platelet (×10^3^) – /µL	242.6±59.4	229.1±65.0	0.08
WBC	11508±2422	12036±2135	0.10
BUN (mg/dl)	16.3± 3.9	16.6± 4.7	0.53
Creatinine (mg/dl)	0.48±0.08	0.46±0.07	0.06
Sodium (mEq/L)	137.4±1.9	137.8± 1.8	0.42
Potassium (mEq/L)	4.1±0.3	4.1±0.2	0.93

WBC: White Blood Cell

Although there were no differences in the Tp–e interval and Tp-e/QTc ratio between the groups, the maximum QTc, minimum QTc, and QTc dispersion values were significantly higher in Group-1 compared to the control group (Table-III). In addition, the P dispersion was greater in the pregnant women of advanced age.

**Table-III:** The electrocardiographic findings of the study population.

	Group-1 (≥35 years)	Group-2 (<35 years)	P-value
Maximum QTc interval (ms)	403.7± 27.8	393.3± 14.6	< 0.001
Minimum QTc interval (ms)	381.8± 23.7	373.8± 13.2	0.002
QTc dispersion (ms)	21.8±8.7	19.5±7.2	0.01
Tp-e interval (ms)	76.3±13.0	74.2±13.0	0.19
Tp-e/QTc ratio	0.18±0.03	0.18±0.03	0.86
P dispersion (ms)	19.5±7.5	17.8±7.2	0.05

In bivariate correlation analysis, the increased dispersion of QTc and P was positively correlated with maternal age (Table-IV).Multiple linear regression analysis showed that the QTc dispersion was independently associated with maternal age (Table-V).

**Table-IV:** Correlation analysis between electrocardiographic findings and clinical characteristics in pregnants.

	Maternal age	BMI
Maximum QTc	r: 0,241 p:<0,001	r: 0,115 p:0,06
Minimum QTc	r:0,192 p:0,001	r: 0,113 p:0,06
QTc dispersion	r: 0,199 p:0,001	r:0,014 p:0,06
Mean QTc	r: 0,225 p:<0,001	r:0,111 p:0,07
Tp-e interval	r: 0,123 p:0,08	r:-0,072 p:0,23
Tp-e/QTc ratio	r: 0,056 p:0,33	r: -0,110 p:0,07
P dispersion	r: 0,132 p: 0,02	r: 0,08 p:0,16

BMI: Body Mass Index

**Table-V:** Results of multivariable analysis of independent predictors of QTc dispersion.

	Unstandardized Coefficients	Standardized Coefficients (ß)	P-value
Maternal age	0.179	0.183	0.003
BMI	128	0.007	0.21

## DISCUSSION

The main findings of our study are that atrial and ventricular repolarization parameters were significantly higher in pregnant women of advanced age compared to younger pregnant women. In addition, we determined that the repolarization parameters of atrial and ventricular increased with increasing age, while only maternal age was predictive of QTc dispersion.

Due to socio-economic improvements and the prolongation of education, women have started becoming pregnant later in life. Therefore; cardiovascular problems such as gynecological and obstetric problems have increased. Briller et al. investigated the specific etiology of maternal deaths where more than 20% of maternal deaths were related to cardiovascular disease such as cardiomyopathy or arrhythmia and more than 25% of these deaths were potentially preventable.^6^ In a study investigating the frequency of arrhythmias, the frequency of arrhythmias increased, mostly ventricular ectopic activity in young healthy pregnancies, particularly among those with palpitation complaints.^7^ Reproductive hormones lay an important role in the onset and progression of arrhythmia such as supraventricular tachycardia and acquired long QT syndrome.^8^,^9^

The electrocardiogram is a common medical tool used for predicting arrhythmogenic risk in clinical practice. The QT interval and its correction by heart rate (QTc), QT interval dispersion, and recently published markers such as the Tp-e interval and Tp-e/QTc ratio have been proposed as markers for predicting the development of malign cardiac arrhythmia and recommended as alternatives for the risk stratification of sudden cardiac death in women with several medical conditions.^10^-^12^

In a study evaluating the risk of cardiac arrhythmia in a preeclampsia (PE) group, the maximum QT, QTc dispersion, Tp-e interval, and Tp-e/QTc ratio values were found to be significantly higher in the PE group than in the healthy pregnant group.^13^ Another study examining the changes in atrial and ventricular repolarization markers during pregnancy found that P dispersion, maximum QTc interval, Tp-e interval and Tp-e/QT ratio were increased in late pregnancy, but still remained within the normal range.^14^ Recently, Braschi et al. demonstrated the increase in ventricular repolarization markers with increased age.^15^ In our study, maximum QTc, minimum QTc, and QTc dispersion values were found to have increased in advanced age pregnancies. In addition, only the maternal age was determined as a predictor of the QT dispersion.

## CONCLUSION

In conclusion, repolarization parameters were increased in advanced-age pregnancies even though they remained in the normal range, which should be explained in follow-up studies that examine whether it is a pathological condition. If this is pathological, arrhythmia risk scorings should be established to reduce mortality and morbidity in advanced-age (≥35 years) with last trimester pregnant women.

### Authors’ Contribution

**MMA:** Conception & design, acquisition of data, analysis, drafting the article, revision of the article, final approval.

**AA:** Conception & design, acquisition of data, final approval.
